# Linezolid Induced Lactic Acidosis: The Side Effect, Clinician Should Be Aware of

**DOI:** 10.7759/cureus.11514

**Published:** 2020-11-16

**Authors:** Savannah Nightingale, Charlotte Austin, Khushboo K Agarwal, Mayurkumar Patel, Mohammad Hossain

**Affiliations:** 1 Internal Medicine, St. George's University School of Medicine, Neptune City, USA; 2 Surgery, Monmouth Medical Center, Long Branch, USA; 3 Internal Medicine, Jersey Shore University Medical Center, Neptune City, USA

**Keywords:** linezolid, lactic acidosis

## Abstract

Linezolid is a synthetic antibiotic that functions through the inhibition of bacterial protein synthesis by binding to ribosomal ribonucleic acid (rRNA). Deliverable in both intravenous and oral form, with a low level of resistance amongst Methicillin-resistant Staphylococcus aureus (MRSA) strains, it is recommended for a wide range of gram-positive infections. We present a case of a male patient who underwent endovascular abdominal aortic aneurysm repair complicated by abdominal sepsis due to bowel ischemia; several days after linezolid therapy was initiated, he presented with signs of lactic acidosis. After excluding other sources such as metabolic, hypoxia, or organ damage, the resulting lactic acidosis was determined to be a side effect of linezolid.

## Introduction

Linezolid is a bacteriostatic compound with a unique method of action. This synthetic antibiotic inhibits bacterial protein synthesis by binding to the bacterial 23S ribosomal ribonucleic acid (rRNA) of the 50S large subunit bacterial/archean ribosome, halting translation of a bacterial protein [1]. Linezolid is not affected by the cytochrome P450 system and does not require dose modification in patients with liver dysfunction or kidney failure. This antibiotic has a relatively mild side effect profile [1,2]. The most notable adverse events are diarrhoea, nausea, headache, and thrombocytopenia, but more severe side effects, including peripheral neuropathy, serotonin syndrome, and lactic acidosis, have been described.

## Case presentation

We present the case of a 67-year-old male with a past medical history of chronic obstructive pulmonary disease, coronary artery disease, hypertension, and chronic kidney disease who underwent elective abdominal aortic aneurysm repair. His initial postoperative course was complicated by ischemic colitis and subsequent abdominal sepsis, resulting in the need for an emergent Hartmann’s procedure. Postoperatively, the patient required intubation and vasopressors and was admitted to the surgical intensive care unit.

The patient had a prolonged course in the ICU, ultimately undergoing a tracheostomy. He was treated with numerous antibiotics throughout his hospital stay to treat, at various instances, intra-abdominal sepsis, bacteremia, and pneumonia. After cultures taken from the patient’s sputum grew Staphylococcus aureus, the patient was started on 600 mg of linezolid every 12 hr for a seven-day course. 

At this point in the patient’s hospital course, the measured lactate level was 2.7 mmol/L. The lactate level steadily increased, peaking at 7.7 mmol/L six days after the initiation of linezolid. The lactate level continued to rise even though the patient was able to be completely weaned from vasopressors, decreasing the likelihood that sepsis was the cause. His renal function throughout this time was downtrending with a creatinine of 1.97 mg/dL on the sixth day of linezolid. His creatinine level on the day of initiation of the drug was 2.53 mg/dL. His liver function throughout this time remained stable. This patient also had a down-trending white blood cell (WBC) count of 14.2 with 10.4% bands. A consulting nephrologist suggested linezolid as a potential source. After linezolid was stopped, the patient’s lactate level steadily decreased until it normalized a few days later.

## Discussion

Linezolid induced lactic acidosis has been described in several case reports in the literature [3-8]. One small retrospective study found that 6.8% of patients taking linezolid developed lactic acidosis unexplained by other factors [3]. A proposed mechanism is that linezolid may interfere with mitochondrial adenosine triphosphate (ATP) synthesis. By binding the A-site at the peptidyl-transferase centre, it may prevent the synthesis of mitochondrial-derived respiratory complexes (I, III, IV, V), which are essential for the electron transport chain and oxidative phosphorylation [4]. 

Several sources have suggested that a prolonged course of linezolid increases a patient's risk of developing lactic acidosis. It's proposed that this is due to increased exposure of mitochondrial proteins to toxic effects of the drug. A retrospective study comparing Linezolid and Teicoplanin noted that each 'definite' case of induced lactic acidosis occurred in patients being treated for a minimum duration of four weeks [3]. Another paper reported three pediatric cases of linezolid induced lactic acidosis; in two of these cases, the lactic acidosis was seen after cumulative usage of more than 31 days [5]. The Infectious Diseases Society of America Emerging Infections Network, and noted that of 29 reported cases of linezolid induced lactic acidosis, a vast majority were in patients on a four-week course or greater [8]. Though most reports in the literature show a correlation between long courses of linezolid and lactic acidosis, our patient was initially prescribed a seven-day course and demonstrated an increased lactate level only three days into treatment.

Linezolid induced lactic acidosis may be more common in patients with renal or hepatic insufficiency, but the association between these things is not clear. A study conducted on an end-stage renal disease patient concluded that it is not a predisposing condition for the development of this side effect [6]. Conversely, the analysis of pediatric patients suggested that an increased creatinine before the initiation of linezolid treatment may have contributed to the development of lactic acidosis. While renal dysfunction does result in the accumulation of inactive metabolites of linezolid, however, it is unclear whether those byproducts contribute to the development of lactic acidosis [7]. There may also be a relationship between hepatic dysfunction and the increased incidence of lactic acidosis. Each pediatric patient with lactic acidosis suffered from some variation of hepatic insufficiency, though the connection between the two is not clear. Another case report mentioned above suggested a possible connection between liver disease and lactic acidosis. Similarly, this study was unable to provide concrete conclusions regarding the connection as they did not include enough patients with chronic liver disease in the study. This possible contributing factor to the development of lactic acidosis merits further study as this drug is not renally or hepatically dosed.

Our patient did not suffer from hepatic insufficiency but had chronic kidney disease (stage 4). However, his creatinine remained stable throughout his hospital course.

## Conclusions

Lactic acidosis is a poorly understood and less often described side effect of Linezolid. This can be seen even in patients treated with short courses of Linezolid. This case reported highlights the importance and necessity to further examine the complete side effect profile of this broad-spectrum antibiotic. This patient underwent tremendous hardship throughout his hospital course from undergoing surgery to developing sepsis. It is impossible to pinpoint the source of his rising lactate level to solely the use of this drug, however, it was not able to be ruled out as a cause. As evidenced in this case report, more research is warranted to determine the mechanism of this adverse side effect and ultimately the impact this drug can have regarding the success of a patient’s outcome.
